# The Atrophic Effect of 1,25(OH)_2_ Vitamin D_3_ (Calcitriol) on C2C12 Myotubes Depends on Oxidative Stress

**DOI:** 10.3390/antiox10121980

**Published:** 2021-12-12

**Authors:** Tommaso Raiteri, Ivan Zaggia, Simone Reano, Andrea Scircoli, Laura Salvadori, Flavia Prodam, Nicoletta Filigheddu

**Affiliations:** 1 Department of Translational Medicine, University of Piemonte Orientale, 28100 Novara, Italy; tommaso.raiteri@uniupo.it (T.R.); ivanzaggia@gmail.com (I.Z.); simone.reano@med.uniupo.it (S.R.); andrea.scircoli@uniupo.it (A.S.); laura.salvadori@uniupo.it (L.S.); 2 Department of Health Sciences, University of Piemonte Orientale, 28100 Novara, Italy; flavia.prodam@med.uniupo.it

**Keywords:** skeletal muscle atrophy, aging, cachexia, sarcopenia, vitamin D receptor (VDR), mitochondrial respiration, oxidative stress, ROS production

## Abstract

Dysfunctional mitochondrial metabolism has been linked to skeletal muscle loss in several physio-pathological states. Although it has been reported that vitamin D (VD) supports cellular redox homeostasis by maintaining normal mitochondrial functions, and VD deficiency often occurs in conditions associated with skeletal muscle loss, the efficacy of VD supplementation to overcome muscle wasting is debated. Investigations on the direct effects of VD metabolites on skeletal muscle using C2C12 myotubes have revealed an unexpected pro-atrophic activity of calcitriol (1,25VD), while its upstream metabolites cholecalciferol (VD3) and calcidiol (25VD) have anti-atrophic effects. Here, we investigated if the atrophic effects of 1,25VD on myotubes depend on its activity on mitochondrial metabolism. The impact of 1,25VD and its upstream metabolites VD3 and 25VD on mitochondria dynamics and the activity of C2C12 myotubes was evaluated by measuring mitochondrial content, architecture, metabolism, and reactive oxygen species (ROS) production. We found that 1,25VD induces atrophy through protein kinase C (PKC)-mediated ROS production, mainly of extramitochondrial origin. Consistent with this, cotreatment with the antioxidant N-acetylcysteine (NAC), but not with the mitochondria-specific antioxidant mitoTEMPO, was sufficient to blunt the atrophic activity of 1,25VD. In contrast, VD3 and 25VD have antioxidant properties, suggesting that the efficacy of VD supplementation might result from the balance between atrophic pro-oxidant (1,25VD) and protective antioxidant (VD3 and 25VD) metabolites.

## 1. Introduction

The progressive loss of muscle strength and functionality that characterizes different physiological (aging) and pathological (sarcopenia, cachexia, idiopathic chronic fatigue, myasthenia gravis) statuses is thought to be due to alterations in mitochondria, including reduced mitochondrial content, impaired oxidative capacity, and increased oxidative damage [1,2,3,4,5].

As a deficit of vitamin D is often associated with these muscle-affecting conditions, vitamin D supplementation has been proposed as a potential treatment to improve muscle strength, albeit with conflicting results. In particular, supplementation of vitamin D appears to be efficacious in restoring muscle function in elderly but not in cachectic muscle [6,7,8,9,10,11,12]. Several works have investigated the effects of vitamin D on mitochondrial function in muscle-derived cells. In particular, the treatment of both human primary and C2C12 myoblasts with 1α,25-dihydroxyvitamin D3 (1,25VD) increases mitochondrial function [13,14,15]. This activity of 1,25VD on mitochondria is even effective in mitigating the dysfunctional metabolism induced by the treatment of myoblasts with conditioned medium derived from cancer cells [16]. Additionally, 1,25VD increases mitochondrial respiration in myotubes derived from the differentiation of both human and C2C12 myoblasts [14,17]. Notably, in both C2C12 myoblasts and myotubes and human myoblasts, the silencing of the vitamin D receptor (VDR) results in a significant reduction in mitochondrial respiration [13,15]. Accordingly, the reduction in mitochondrial respiration in human myoblasts upon treatment with cholecalciferol (VD3) or 25-hydroxyvitamin D3 (25VD) led to the hypothesis that the increase in mitochondrial oxygen consumption rate (OCR) depends on the high binding affinity of 1,25VD for VDR relative to the lower affinities of other metabolites [13].

However, despite the evidence correlating vitamin D deficiency with muscular impairment and the undisputed importance of the VD/VDR system for skeletal muscle health, treatment of C2C12 myotubes with 1,25VD negatively impinges on myotube size [11,18]. In contrast, the upstream metabolites of 1,25VD, namely VD3 and 25VD, have an anti-atrophic activity on the same in vitro cellular model [18,19]. 

Since mitochondrial dysfunction can induce muscle atrophy in several conditions [20], in the present study, we investigated the effects of 1,25VD and its upstream metabolites VD3 and 25VD on mitochondrial function and organization in C2C12 myotubes.

## 2. Materials and Methods

### 2.1. Reagents

VD3, 25VD, and 1,25VD were purchased from Merck Life Sciences (Milan, Italy) and dissolved in ethanol. Protein kinase C (PKC) inhibitor (GÖ6850) and c-Jun N-terminal kinase (JNK) inhibitor (SP600125) were from Cayman Chemical (Ann Arbor, MI, USA). N-Acetyl-L-Cysteine (NAC), MitoTracker Red CMXRos, and CellROX R Deep Red Reagent were from Invitrogen (Thermo Fisher Scientific, Waltham, MA, USA). JC-1 was from Adipogen Life Sciences (Liestal, Switzerland). Anti-LC3B antibody was from Proteintech (Rosemont, IL, USA), antitubulin antibody was from Santa Cruz Biotechnology (Dallas, TX, USA), and anti-VDAC and antiphospho-(Ser) PKC substrates antibodies were from Cell Signaling Technology (Danvers, MA, USA). MiR05 was purchased from Oroboros Instruments (Innsbruck, Austria). All other reagents, unless otherwise stated, were from Merck Life Sciences (Milan, Italy). 

### 2.2. Cell Culture and Myotube Analysis

C2C12 myoblasts (ECACC, Porton Down, Salisbury, UK) were grown at low density in Dulbecco’s Modified Eagle Medium (DMEM, Gibco, Thermo Fisher Scientific, Waltham, MA, USA) supplemented with 10% fetal bovine serum (FBS, Gibco, Thermo Fisher Scientific, Waltham, MA, USA), 100 U/mL penicillin, 100 μg/mL streptomycin, and 0.25 μg/mL antimycotic in a humidified 5% CO_2_ incubator at 37 °C. To induce differentiation, cells were allowed to become confluent, and the medium was switched to differentiation medium (DM), consisting of DMEM supplemented with 2% horse serum (GE Healthcare BioSciences, Uppsala, Sweden), penicillin, streptomycin, and antimycotic as described above. Unless otherwise specified, myotubes were in treated serum-free medium after at least five days of differentiation. Control cells were treated with 0.1% ethanol. Myotube diameters were measured with JMicroVision software (v. 1.3.4, University of Geneva, Switzerland) as previously described [21]. For every experiment assessing myotube diameters, at least 10 myotubes for each field, five different fields for each replicate, and three technical replicates for each treatment were measured. Displayed data are the average of three independent experiments.

### 2.3. DNA Extraction and Analysis

DNA was extracted from myotubes using the NucleoSpin Tissue purification kit (Macherey-Nagel, Düren, Germany) according to the manufacturer’s instructions. The isolated DNA was eluted in 60 µL of elution buffer. To investigate changes in mitochondria number, real-time PCR was performed with the StepOnePlus Real-time PCR System (Applied Biosystems, Thermo Fisher Scientific, Waltham, MA, USA), using the following TaqMan probes (Thermo Fisher Scientific, Waltham, MA, USA): *Rps18* (Mm00507222_s1) and *Cox2* (Mm03294838_g1). 

### 2.4. RNA Extraction and Analysis

Total RNA from myotubes was extracted by RNAzol (Merck Life Sciences, Milan, Italy). The RNA was retrotranscribed with high-capacity cDNA Reverse Transcription Kit (Applied Biosystems, Thermo Fisher Scientific, Waltham, MA, USA), and real-time PCR was performed with the StepOnePlus Real-time PCR System (Applied Biosystems, Thermo Fisher Scientific, Waltham, MA, USA), using the following TaqMan probes (Thermo Fisher Scientific, Waltham, MA, USA): *Opa1* (Mm00453873_m1), *Mff* (Mm01273401_m1), *Mfn2* (Mm00500120_m1), *FbxO32* (Atrogin-1, Mm00499523_m1), *Trim63* (MuRF-1, Mm01185221_m1), and *Gusb* (Mm01197698_m1).

### 2.5. Mitochondrial Membrane Potential Analysis 

Mitochondrial membrane potential in C2C12 myotubes was assessed using the JC-1 mitochondrial membrane potential assay kit (Adipogen Life Sciences, Liestal, Switzerland), according to the manufacturer’s instructions. Briefly, 10 µg/mL JC-1 was added to the culture medium and gently mixed. Cells were then incubated in a CO_2_ incubator at 37 °C for 15 min. Images were acquired with a fluorescence microscope (EVOS™ XL Core Imaging System, Thermo Fisher Scientific, Waltham, MA, USA). Red emission of the dye represented a potential-dependent aggregation of JC-1 in the mitochondria. Conversely, green fluorescence appearing in the cytosol after mitochondrial membrane depolarization represented the monomeric form of JC-1. The average intensity of red and green fluorescence was measured using ImageJ software, and the ratio of JC-1 aggregate (red) to monomer (green) intensity was then calculated. For every experiment assessing mitochondrial membrane potential, at least five myotubes in each field, five different fields for each replicate, three technical replicates for each treatment were measured. The final data are the average of three independent experiments.

### 2.6. Determination of Reactive Oxygen Species (ROS) Production

To measure cellular ROS production, C2C12 myotubes were stained with CellROX R Deep Red Reagent (Thermo Fisher Scientific, Waltham, MA, USA) for 30 min at 37 °C and washed with PBS. Fluorescent images were taken using fluorescence microscope EVOS™ XL (Thermo Fisher Scientific, Waltham, MA, USA), and the mean fluorescence signal intensity was measured using ImageJ (v. 1.53j, National Institutes of Health, USA). For every experiment assessing cellular oxidative stress, at least five myotubes in each field, five different fields for each replicate, three technical replicates for each treatment were measured. The final data are the average of three independent experiments.

### 2.7. Mitochondrial Morphology and Mitophagy Evaluation 

Fluorescent microscopy was performed to identify variations in both mitochondrial morphology and mitophagy. For mitochondrial network visualization, C2C12 myotubes were incubated with 100 nM MitoTracker Red CMXRos (Thermo Fisher Scientific, Waltham, MA, USA) for 45 min at 37 °C, 5% CO_2_. To analyze mitophagy, cells were washed in PBS, fixed with 4% paraformaldehyde for 10 min, and permeabilized for 5 min with 0.2% Triton X-100 (Merck Life Sciences, Milan, Italy). Blocking was then performed with 4% bovine serum albumin for 30 min. Subsequently, cells were incubated with a primary antibody directed against LC3B (microtubule-associated protein 1 light chain-3B) overnight at 4 °C and followed by the appropriate Alexa Fluor Dye-conjugated secondary antibody (488 antirabbit 1:400; Thermo Fisher Scientific, Waltham, MA, USA) for 1 h at room temperature. Nuclei were counterstained with DAPI (1:100, Thermo Fisher Scientific, Waltham, MA, USA), and images were acquired with a Leica dm 5500b fluorescence microscope (Leica, Wetzlar, Germany) equipped with Leica Application Suite X software (v.3.7.4.23463, Leica, Wetzlar, Germany), using a 40× objective. For every experiment assessing mitochondrial morphology and mitophagy, at least two myotubes in each field, five different fields for each replicate, three technical replicates for each treatment were measured. The final data are the average of three independent experiments.

Mitochondrial morphology was analyzed using the Mitochondrial Network Analysis tool (MiNA, v.3.0.1), a macro tool developed for ImageJ, as previously described [22]. Briefly, fluorescence images were processed removing the background and then skeletonized, and the resulting skeleton was analyzed using the Analyze Skeleton plugin included in the ImageJ software.

To quantify mitophagy, Pearson’s correlation coefficient of the fluorescent signals in both channels was calculated through the JACoP plugin [23]. This coefficient describes the correlation between the intensity distribution or pattern overlap in two channels in terms of a least-squares fit. This value can be between −1 and 1, and *R* = 1 indicates a complete correlation between the two channels. Finally, Pearson’ s coefficient indicates the overlap of the signals and represents the degree of colocalization.

### 2.8. Co-Immunoprecipitation Assay

For the co-immunoprecipitation assay, 15 min before the end of the indicated treatments, 1 mM of the chemical cross-linker 3-3′-dithiodipropionic acid di-(N-hydroxysuccinimide ester) was added. Cells were resuspended in ice-cold 4-(2-hydroxyethyl)-1-piperazineethanesulfonic acid (HEPES)-buffered saline (HBS: 150 mM NaCl, 20 mM HEPES) supplemented with protease inhibitor cocktail and lysed by passing through a 29-gauge needle 30 times. Nuclei and cell debris were spun down at 500× *g* for 5 min. Protein were quantified by BCA assay (ThermoFisher Scientific, Waltham, MA, USA), and 250 μg of proteins from each lysate were incubated for 4 h with primary anti-LC3B antibody (1:250) on a rotating wheel. To capture the immunocomplexes, 20 μL of Protein G sepharose Fast Flow were added to each sample and left under rotation overnight at 4 °C. After two washes with HBS, immunocomplexes were collected by centrifugation and eluted in 30 μL of sample buffer (2% sodium dodecyl sulfate, 150 mM dithiothreitol, and 0.01% bromophenol blue) at 95 °C for 10 min. Finally, the association LC3B-VDAC was evaluated by western blotting. First, 15 μg of the whole cell lysate (input) and all the immunoprecipitated fraction were separated by 15% SDS-PAGE and transferred to polyvinylidene difluoride filters (PVDF) (Hybond-P; GE Healthcare, Little Chalfont, Buckinghamshire, UK). Membranes were then saturated with 4% bovine serum albumin (BSA), incubated with anti-LC3B (1:600), anti-VDAC (1:1000), or antitubulin (1:1000) antibodies overnight, washed with Tris-buffered saline (TBS) 0.1% Tween, and incubated with the appropriate secondary antibody (1:3000; Invitrogen, Thermo Fisher Scientific, Waltham, MA, USA) for 1 h at room temperature. Specific signals were visualized with Western Lightning Chemiluminescence Reagent Plus (PerkinElmer Life and Analytical Sciences, Waltham, MA, USA), and acquired with ChemiDoc Touch (Bio-Rad, Hercules, CA, USA).

### 2.9. Western Blotting

At the end of the indicated treatments, cells were washed in ice-cold PBS and solubilized with a lysis buffer containing 1% Triton X-100, 0.1% sodium deoxycholate, 0.1% sodium dodecyl sulfate, 1 mM EDTA, 1 mM EGTA, 50 mM NaF, 160 mM NaCl, 20 mM Tris-HCl, pH 7.4, and supplemented with protease inhibitor cocktail. Lysates were stirred at 4° C for 15 min and centrifuged at 15,000× *g* for 15 min at 4° C. Protein concentration was determined by BCA protein assay kit. Proteins (20 μg protein/lane) were separated by 10% SDS-PAGE and transferred to PVDF. Membranes were saturated with 4% BSA, incubated with the antiphospho-(Ser) PKC substrates and antitubulin antibodies (1:1000) overnight, washed with TBS 0.1% Tween, incubated with the appropriate secondary antibody (1:3000) for 1 h at room temperature, visualized with Western Lightning Chemiluminescence Reagent Plus, acquired with ChemiDoc Touch, and analyzed with ImageLab (Bio-Rad, Hercules, CA, USA).

### 2.10. Intact Cell Respiration Using High-Resolution Respirometry

We determined cellular respiration using an Oroboros oxygraph-2K high-resolution respirometer (Oroboros Instruments, Innsbruck, Austria) and substrate, uncoupler, inhibitor, titration (SUIT) protocols [24,25,26]. To assess mitochondrial respiration in intact cells, we used the SUIT-003_O2_ce_D012 protocol, as recommended by the manufacturer of the Oroboros instrument. At the end of the treatments, C2C12 myotubes were trypsinized, centrifuged at 300× *g* for 5 min, resuspended in mitochondrial respiration medium MiR05 (0.5 mM EGTA, 3.0 mM MgCl_2_·6H_2_O, 60 mM potassium lactobionate, 20 mM taurine, 10 mM KH_2_PO_4_, 20 mM HEPES, 110 mM sucrose, 1 g/L bovine serum albumin, pH 7.1) and transferred to the chambers of the Oroboros oxygraph. Control and treated samples were assessed simultaneously. After initial stabilization of O_2_ flux, pyruvate (5 mM) was used to sustain TCA-linked respiration in MiR05 medium. ATP synthetase inhibitor, oligomycin (Omy), was added at 5 nM final concentration, and oxygen consumption was quantified to determine the oligomycin-sensitive and -insensitive respiration. Protonophore (H^+^ ionophore) and uncoupler of oxidative phosphorylation, carbonyl cyanide-p-trifluoromethoxyphenylhydrazone (FCCP) (U) was then added at 0.5 μM increments to achieve maximum respiration to quantify maximum respiratory capacity. This was followed by rotenone (Rot) 500 nM final concentration, to inhibit complex I of the electron transport chain (ETC), and then 2.5 μM antimycin A (Ama), which inhibits complex III, was added to determine the nonmitochondrial respiration (ROX). Oxygen consumption rates were calculated using accompanying software (DatLab7, Oroboros, Innsbruck, Austria) as described earlier [24]. Rates of O_2_ consumption (flux) were normalized to total protein content. Briefly, at the end of the experimental procedure, the cellular suspension from the two chambers was centrifuged at 1000× *g* for 5 min. The cellular pellet was lysed in 200 µL of lysis buffer (10 mM HEPES, 60 mM KCl, 1 mM EDTA, 0.075% NP40, 1 mM DTT) and then centrifuged at 15,000× *g* for 15 min at 4 °C. The concentration of the protein in the supernatant was measured with Bradford Reagent (Merck Life Sciences, Milan, Italy). 

### 2.11. Mitochondrial Oxidative Phosphorylation (OXPHOS) in Permeabilized Myotubes

To quantify mitochondrial function in permeabilized myotubes, we performed high-resolution respirometry using the SUIT-008_O2_ce-pce_D025 protocol designed by Oroboros Instruments to standardize the evaluation of OXPHOS and electron transfer capacities linked to the complex I and II. Briefly, C2C12 myotubes were trypsinized, centrifuged at 300× *g* for 5 min, and resuspended in mitochondrial respiration medium MiR05. After the transfer in the oxygraph, cell permeabilization by 10 µg/mL of digitonin was followed by the addition of mitochondrial substrates malate (2 mM) and pyruvate (5 mM) to generate NADH, substrate for complex I. To maintain the oxidation of pyruvate to acetyl CoA via pyruvate dehydrogenase, malate was added simultaneously as a source of oxaloacetate to metabolize acetyl CoA to citrate. Leakage respiration was measured in the presence of pyruvate and malate without ADP. The addition of ADP at a saturating concentration (2.5 mM) was used to quantify OXPHOS capacity, that is, the capacity of oxidation (electron transport down the gradient along the ETC) and ATP synthesis of complex I. The integrity of the mitochondrial membrane was tested by adding 10 µM cytochrome c. Malate, pyruvate, and glutamate (10 mM), provided as complex I substrates, were followed by succinate (10 mM) as a complex II substrate to quantify the OXPHOS capacity of both complexes I and II. The contribution of complex II to the OXPHOS was obtained as the difference of oxygen flux before and after the addition of succinate. Oxidation and phosphorylation were uncoupled with 0.5 µM FCCP to measure maximal respiration. The uncoupled rate of oxygen consumption of complex I (the rotenone-sensitive rate) was determined by the absolute decrease from the maximum uncoupling rate of all substrates. The rotenone-insensitive rate is the uncoupled rate of oxygen consumption of complex II. After the injection of antimycin, the uncoupled oxidation rate of complex IV was calculated by subtracting the azide-insensitive rate from the tetramethyl phenylene diamine (TMPD, 0.5 mM) + ascorbate (2 mM) rate. Oxygen consumption rates were calculated using accompanying software (DatLab7, Oroboros, Innsbruck, Austria) as described earlier [24] and normalized to protein total content.

### 2.12. Mitochondrial ROS Production

Hydrogen peroxide (H_2_O_2_) production was measured in the presence of horseradish peroxidase (HRP) and Amplex Ultrared Reagent (Invitrogen, Thermo Fisher Scientific, Waltham, MA, USA), which react in a 1:1 stoichiometry with H_2_O_2_ to produce highly fluorescent resorufin. H_2_O_2_ production rates were measured in the oxygraphic chamber, multiplexed with measurements of oxygen consumption using the SUIT-006_AmR_mt_D048 protocol, as recommended by Oroboros Instruments (Innsbruck, Austria). Superoxide dismutase (5 U/mL), horseradish peroxidase (1 U/mL), and Amplex Ultra Red (10 µM) were added into the oxygraphic chambers containing C2C12 myotubes. After permeabilization with digitonin (10 µg/mL), pyruvate (5 mM) and malate (2 mM) were added to evaluate the leakage state (LEAK), while the following supplementation of ADP (2.5 mM) induced the oxygen and H_2_O_2_ flux relative to the maximum OXPHOS capacity. The LEAK state dependent on the ATP-synthase inhibition was obtained with the addition of 5 µM oligomycin. The fluorescent signal was adjusted for background auto-oxidation and calibrated to a standard curve. Rates of H_2_O_2_ production (flux) were normalized to protein total content.

### 2.13. Statistical Analysis

The investigators quantifying the experimental outcomes were blind to the treatments, and the statistic evaluation of the experimental data was performed by another investigator not directly involved in data collection and parameters measurement.

Data are presented as the mean ± SEM. Outliers in the measurements were identified by mean of the interquartile range (IQR), as either below Q1 − 1.5 IQR or above Q3 + 1.5 IQR and excluded from the analysis. The variation among groups was evaluated using Student’s t test or one-way ANOVA test followed by Tukey’s multiple comparisons test, as appropriate. Statistical significance was assumed for *p* < 0.05. All statistical analyses were performed with GraphPad Prism 8 (GraphPad Software, San Diego, CA, USA).

## 3. Results

### 3.1. Opposite Effects of 1,25VD and VD3 on Mitochondrial Membrane Polarization in C2C12 Myotubes 

**Figure 1:**
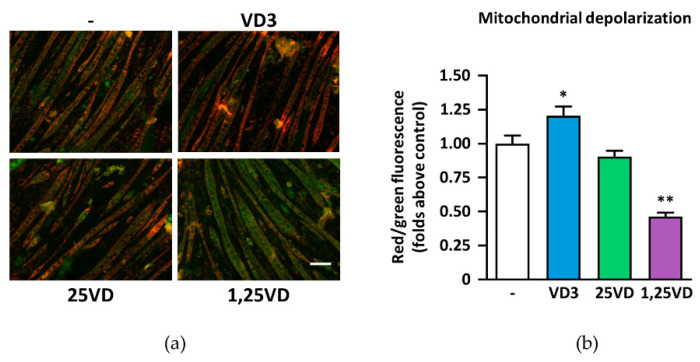
Mitochondrial membrane depolarization induced by 1,25VD. (**a**) Representative images of fluorescence of JC-1 in C2C12 myotubes treated with 100 nM cholecalciferol (VD3), 25-hydroxyvitamin D3 (25VD), or 1,25-dihydroxyvitamin D3 (1,25VD) for 24 h in serum-free medium, scale bar 100 µm; (**b**) Quantification of the ratio of red/green fluorescence in treated cells compared to vehicle-treated controls. Data are presented as means ± SEM of three independent experiments; * *p* < 0.05; ** *p* < 0.01 compared to vehicle-treated cells.

To test the hypothesis that 1,25VD impinges on C2C12 mitochondrial function, we investigated its effects on mitochondrial membrane potential. In agreement with the pro-atrophic effects of 1,25VD [18], we observed that 1,25VD induced the loss of mitochondrial membrane potential in C2C12 myotubes, highlighted by a fluorescence red-to-green emission shift of the cationic dyes JC-1 (Figure 1a,b). In contrast, the anti-atrophic metabolites VD3 and 25VD did not cause mitochondrial membrane depolarization, i.e., 25VD had no effects and VD3 even induced hyperpolarization.

### 3.2. VD3, but Not 1,25VD nor 25VD, Affects Mitochondrial Respiration in Intact Myotubes 

**Figure 2:**
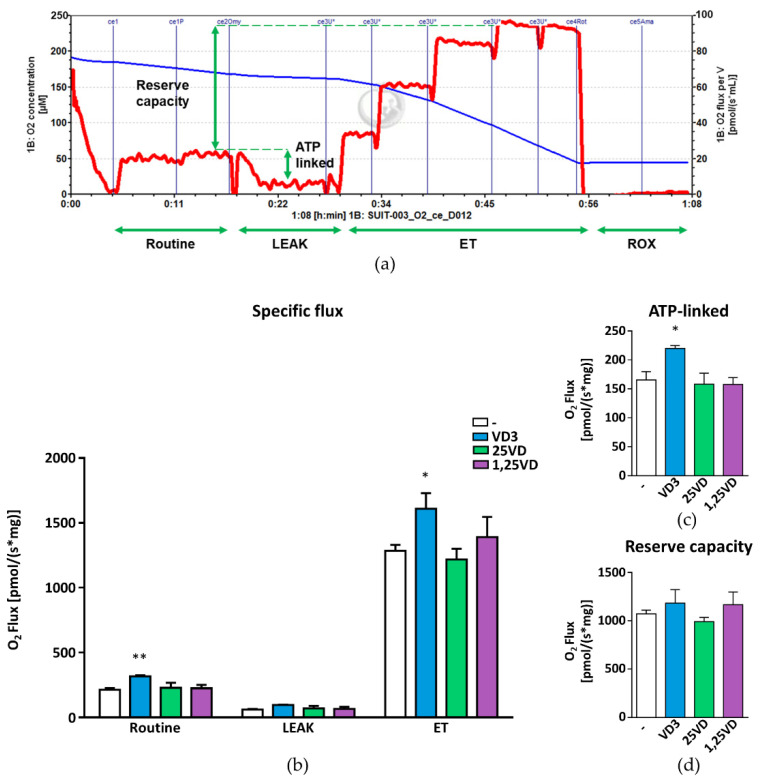
Data showing that 1,25VD does not affect mitochondrial respiration in intact cells. (**a**) Representative tracing of high-resolution respirometry to quantify intact cell respiration of differentiated C2C12 myotubes after 24 h treatment with 100 nM VD3, 25VD, or 1,25VD. (**b**) Oxygen flux in the routine state (Routine); in the leakage state (LEAK) after addition of oligomycin, an inhibitor of ATP synthetase; after the addition of carbonyl cyanide-p-trifluoromethoxyphenylhydrazone (FCCP), an uncoupler of oxidative phosphorylation to induce maximum respiratory capacity (ET). All data are expressed as specific flux, i.e., oxygen consumption normalized to the sample protein content and after nonmitochondrial oxygen flux subtraction (ROX). (**c**) Oxygen consumption linked to ATP production, i.e., oligomycin-sensitive respiration obtained by the subtraction of LEAK from Routine. (**d**) Reserve respiratory capacity obtained by the subtraction of Routine from ET. All data are expressed as mean ± SEM from at least four independent experiments. * *p* < 0.05; ** *p* < 0.01 compared to vehicle-treated cells.

To evaluate the impact of 1,25VD, 25VD, and VD3 on mitochondrial function, we measured oxidative respiration in intact, nonpermeabilized myotubes after 24 h of treatment with the different metabolites. Surprisingly, 1,25VD-induced atrophy was not accompanied by a decrease in oxygen consumption (Figure 2a,b). Likewise, 25VD did not affect mitochondrial respiration in intact cells. Only VD3 significantly increased oxygen consumption in both the basal (routine) and maximal respiration, measured upon treatment with FCCP, a protonophore that uncouples oxidation from phosphorylation. On the other hand, the proton leak, measured as the oxygen consumption when phosphorylation is inhibited by oligomycin, was not affected by VD3. In line with these results, only VD3 increased the ATP-linked respiration (Figure 2c), obtained by subtracting the proton leak from the routine oxygen consumption. In contrast, none of the metabolites affected the reserve respiratory capacity, a critical component of mitochondrial oxidation that can be utilized during states of increased ATP demand (Figure 2d). Of note, in undifferentiated myoblasts, maximal OCR tended to increase upon 1,25VD, in accordance with previously reported data (data not shown).

### 3.3. Mitochondrial Respiration in Permeabilized Myotubes Is Impaired by 1,25VD

**Figure 3:**
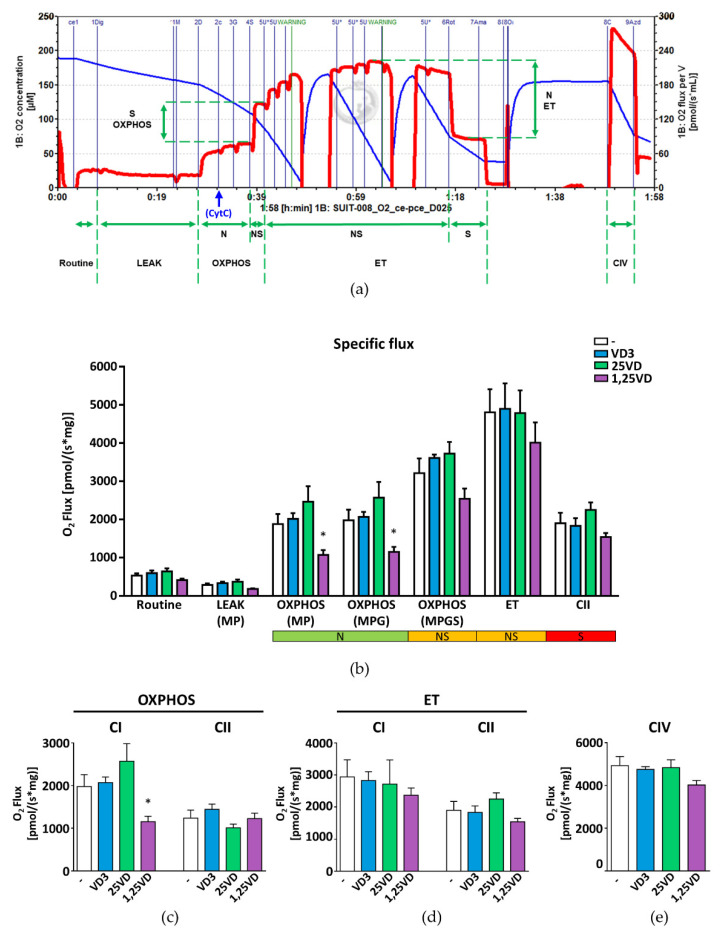
In permeabilized cells, 1,25VD lowers the complex I maximal OXPHOS capacity. (**a**) Representative tracing of high-resolution respirometry to quantify cell respiration of permeabilized C2C12 myotubes treated for 24 h with 100 nM VD3, 25VD, or 1,25VD. (**b**) After the routine state (Routine) and digitonin permeabilization, malate (M) and pyruvate (P) were added to induce the leakage (LEAK) state; ADP induces maximal oxidative phosphorylation (OXPHOS) that, in the presence of the N-linked substrates (N) M, P and glutamate (G), represents the OXPHOS capacity of the complex I. The addition of succinate (S), substrate S-linked (S), results in the cumulative OXPHOS capacity of complexes I and II (NS). FCCP was adjusted to obtain the maximal respiratory capacity (ET) linked to complex I and II, while after rotenone the ET is linked only to the complex II (CII). All data are expressed as specific flux, i.e., oxygen consumption normalized to the sample protein content and after nonmitochondrial oxygen flux subtraction (ROX). (**c**) Complex I OXPHOS capacity in the presence of M, P and G, and complex II OXPHOS capacity obtained by the subtraction of CI OXPHOS capacity from CI + CII OXPHOS capacity (M, P, G and S); (**d**) maximal respiratory capacity of complex I (ET CI) obtained by the subtraction of ET CII from ET and maximal respiratory capacity of complex II (ET CII) obtained after the rotenone addition; (**e**) complex IV activity. All data are expressed as mean ± SEM from at least three independent experiments. * *p* < 0.05 compared to vehicle-treated cells.

To better appreciate potential minimal effects of vitamin D metabolites, we assessed mitochondrial respiration in permeabilized myotubes, a system more similar to isolated mitochondria. To dissect the contribution of the single complexes to mitochondrial respiration and identify the specific step(s) at which the administration of each metabolite modifies it, we performed the SUIT-008_O2_ce-pce_D025 protocol on myotubes treated with VD3, 25VD, and 1,25VD for 24 h. At the end of treatments, myotubes were permeabilized with digitonin. To ensure that permeabilization did not alter mitochondrial integrity, we injected cytochrome c (CytC) and monitored the O_2_ flux before and after the injection. As shown in Figure 3a, cytochrome c addition did not alter the O_2_ flux, indicating that the mitochondrial membranes were intact. The ADP-stimulated oxygen consumption in the presence of malate and pyruvate (MP) and malate, pyruvate, and glutamate (MPG), substrates of complex I, represents the maximal oxidative phosphorylation capacity (OXPHOS) of complex I and was significantly reduced by 1,25VD treatment (Figure 3b,c). In contrast, 25VD promoted a slight, although not significant, increase of complex I OXPHOS, while VD3 had no effect. The addition of succinate (S, substrate of complex II) to MPG produces the maximal OXPHOS of the combination of complexes I and II. None of the metabolites significantly altered the O_2_ flux related to the combined OXPHOS of complexes I and II, despite the tendency of 1,25VD to reduce it. Coherently, the OXPHOS capacity of complex II was not significantly affected by the treatments (Figure 3c). Likewise, none of the treatments could alter the maximal respiratory capacity (ET) measured in response to titration of the uncoupler FCCP (Figure 3b), both relatively to complex I and complex II (Figure 3d), nor the complex IV function, measured as azide-sensitive oxidation of the electron donor to cytochrome c TMPD (Figure 3e).

### 3.4. Mitochondria Quantity and Mitochondrial Network Morphology Are Unaffected by 1,25VD

**Figure 4:**
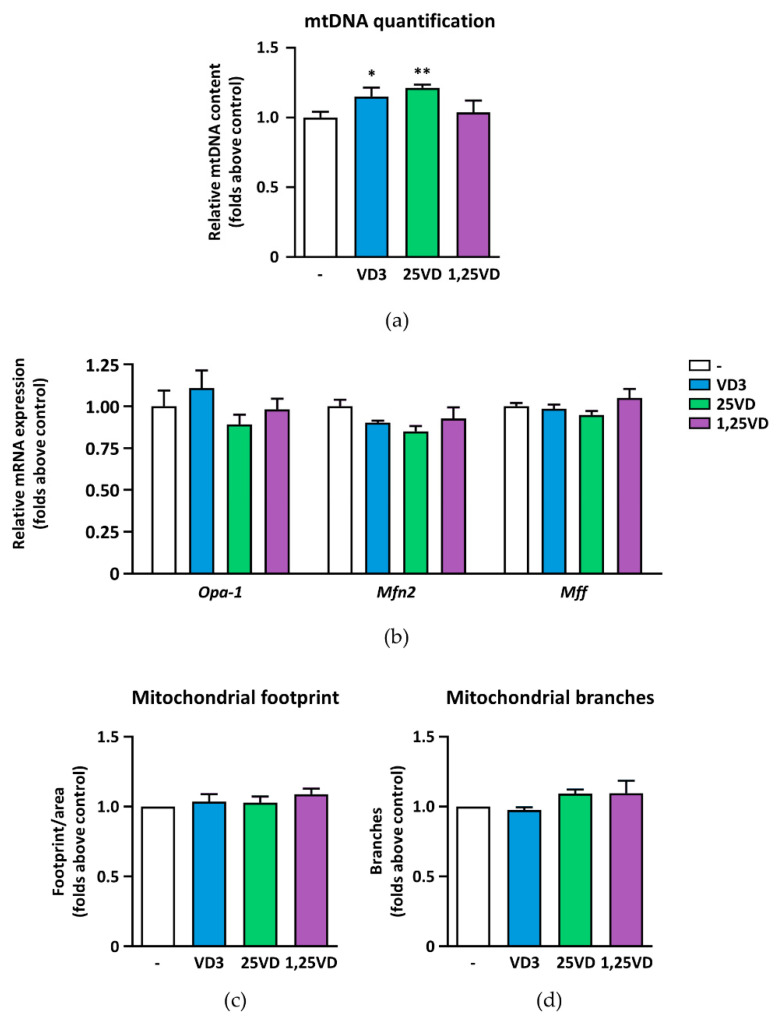
Effects of VD metabolites on mitochondrial content and morphology. (**a**) After treatment with 100 nM VD3, 25VD, and 1,25VD for 24 h in serum-free medium, mitochondrial DNA content in C2C12 myotubes was quantified by measuring, by real-time PCR, the expression of mitochondrial DNA gene *Cox2* normalized on the genomic DNA gene *Rps18*; (**b**) the expression of fusion and fission regulator genes were assessed by real-time PCR using *Gusb* as the housekeeping gene; (**c**) mitochondrial footprint and (**d**) branches in MitoTracker Red-stained mitochondrial network with the MiNA plugin for ImageJ. Data are presented as means ± SEM of three independent experiments. * *p* < 0.05; ** *p* < 0.01 compared to vehicle-treated cells.

Since altered mitochondrial function could be an indication of changed mitochondrial content, and, in contrast, mitochondriogenesis might be a compensative mechanism for mitochondrial dysfunction, we evaluated if vitamin D metabolites altered mitochondria amount by quantifying mitochondrial DNA (mtDNA). The relative expression of the mtDNA gene *Cox2* normalized on the genomic DNA gene *Rps18* showed no significant differences between 1,25VD-treated myotubes and controls (Figure 4a), indicating that 1,25VD does not affect the number of mitochondria in C2C12 myotubes. In contrast, both VD3 and 25VD induced a modest but significant increase of mitochondrial content.

To maintain metabolic efficiency and to compensate for stressor stimuli, mitochondria are continuously remodeled by fusion and fission events that modify their network architecture. However, none of the metabolites altered the expression of the genes involved in fusion (*Opa1* and *Mfn2*) and fission (*Mff*) processes (Figure 4b). Accordingly, no changes in mitochondrial footprints and branches were observed (Figure 4c,d).

### 3.5. Mitophagic Flux Is Blocked by 1,25VD 

**Figure 5:**
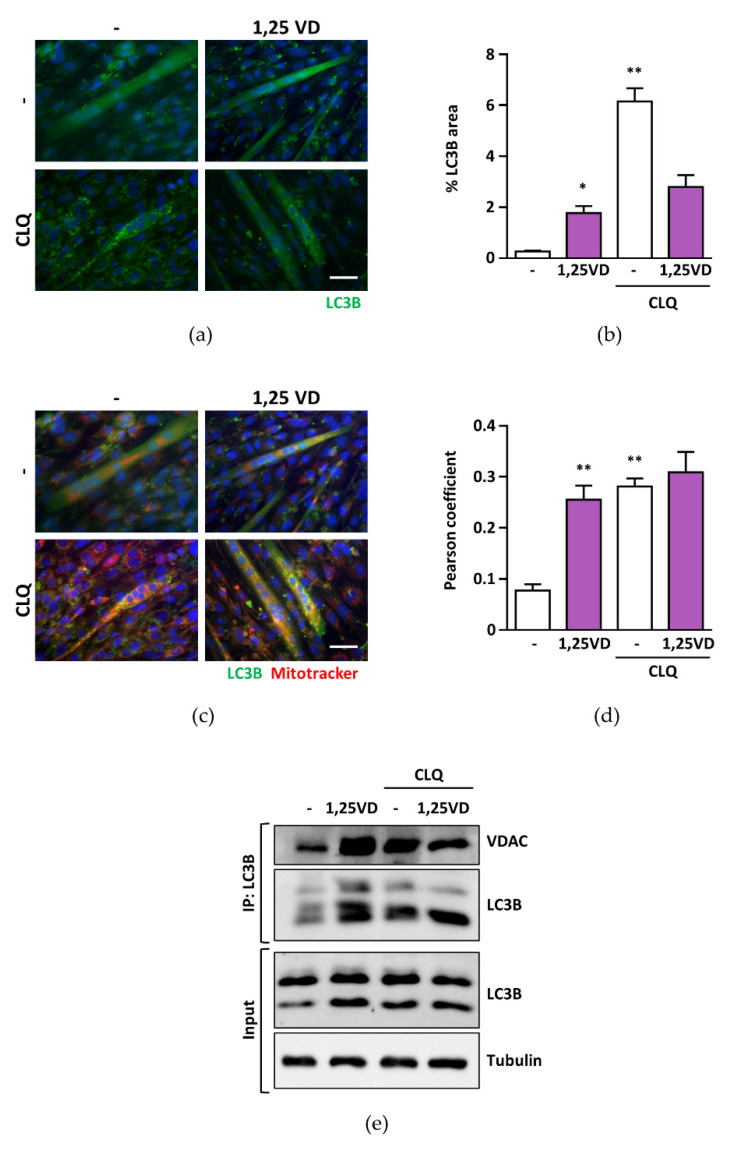
Mitophagic flux is blocked by 1,25VD. C2C12 myotubes were treated in serum-free medium for 24 h with 100 nM 1,25VD in the presence or absence of 10 µM chloroquine (CLQ). (**a**) Representative images of LC3B immunofluorescence, and (**b**) percentage of LC3B puncta above total myotube area. (**c**) To assess the mitophagic process, mitochondria were stained with MitoTracker™ Red CMXRos and fluorescent images were superimposed to the IF of LC3B; (**d**) colocalization of LC3B and MitoTracker, expressed as Pearson’s coefficient, was analyzed with the JACoP plugin for ImageJ. Scale bar 50 µm; data are presented as means ± SEM; * *p* < 0.05; ** *p* < 0.01. (**e**) Co-immunoprecipitation of LC3B and VDAC in C2C12 myotubes in the absence or presence of chloroquine (CLQ). The interaction LC3B-VDAC was analyzed by immunoprecipitating LC3B followed by VDAC immunoblotting. An amount of 15 µg of the lysate used for the immunoprecipitation (input) was immunoblotted with anti-LC3B and antitubulin antibodies.

The lack of changes in mitochondrial dynamics, despite clearly damaged mitochondria, as seen by their membrane depolarization (Figure 1), suggests that additionally, mitochondrial turnover is impaired. To assess the occurrence of the block of the mitophagic flux, we assessed the accumulation of the autophagosomal marker LC3B in C2C12 myotubes in the presence or absence of chloroquine (CLQ), a treatment known to block the autophagic flux (Figure 5a,b), and superimposed the images of the MitoTracker-stained mitochondria (Figure 5c). We observed that 1,25VD increased the accumulation of bona fide mitophagosomes, and that the signal did not further increase upon chloroquine treatment (Figure 5d), suggesting a block of the mitophagic flux. This notion was supported by the facts that 1,25VD treatment increased the amount of mitochondrial VDAC co-immunoprecipitated with autophagosomal LC3B, and that chloroquine was not able to further increase it (Figure 5e).

### 3.6. Reactive Oxygen Species (ROS) Production Is Induced by 1,25VD

**Figure 6:**
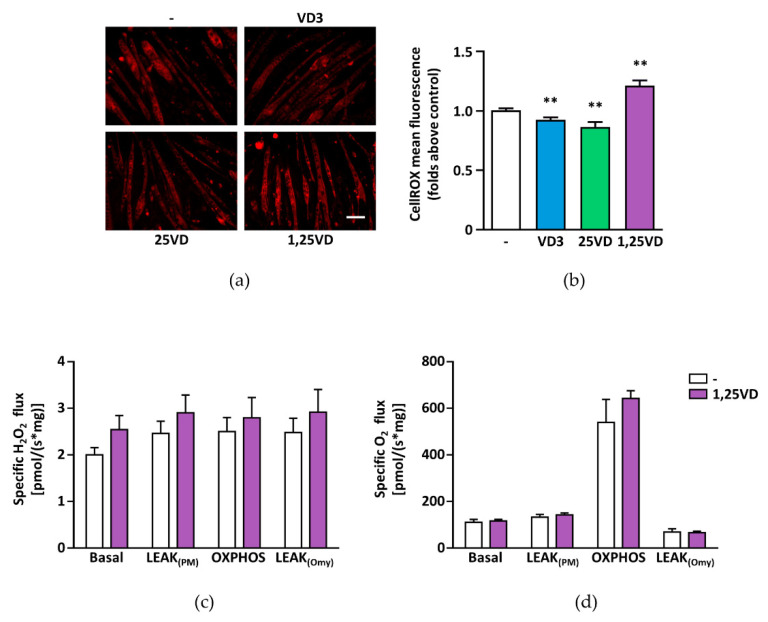
Cholecalciferol (1,25VD)-induced ROS is not caused by mitochondrial ROS production. After treatment with 100 nM VD3, 25VD, and 1,25VD for 24 h in serum-free medium, (**a**) the total ROS production was evaluated by CellROX Deep Red reagent. Scale bar 100 µm. (**b**) The resulting fluorescence was quantified as fluorescence mean intensity of each myotube. (**c**) H_2_O_2_ flux was measured using the Amplex Red fluorescence assay in the chambers of the Oroboros oxygraph; (**d**) multiplexed with measurements of oxygen consumption in four different states obtained by the sequential titrations of substrates, inhibitors, and uncouplers aimed at increasing mitochondrial membrane hyperpolarization and inducing ROS production. Briefly, basal H_2_O_2_ flux is measured before the addition of substrates (pyruvate and malate); LEAK_(PM)_ is the flux after the addition of substrates in the absence of exogenous ADP; OXPHOS is the flux after the addition of ADP, thus stimulating complex I and II respiration; and LEAK_(Omy)_ the flux in the presence of oligomycin that increases membrane polarization. Data are expressed as means ± SEM of three independent experiments; ** *p* < 0.01 compared to vehicle-treated cells.

As the effects of 1,25VD on mitochondria and myotube diameters are divergent from those of VD3 and 25VD (Figure 1, Figure 2 and Figure 3 and [18,19]), we assessed the effect of these metabolites on ROS production in C2C12 myotubes. We observed that 1,25VD caused a significant increase of fluorescence in CellROX-loaded cells, while both VD3 and 25VD lowered the basal levels (Figure 6a,b), suggesting that 1,25VD might induce oxidative stress in C2C12 myotubes, whereas VD3 and 25VD might act as antioxidants.

Although mitochondria are an important source of cellular ROS, the 1,25VD-induced increase in ROS only partially originated from mitochondria, as 1,25VD treatment induced only a slight increase in H_2_O_2_ flux in permeabilized myotubes (Figure 6c). Also, no significant differences of concomitant oxygen consumption were monitored (Figure 6d).

### 3.7. Treatment with Antioxidant Abrogates 1,25VD-Induced Atrophy in C2C12 Myotubes

**Figure 7:**
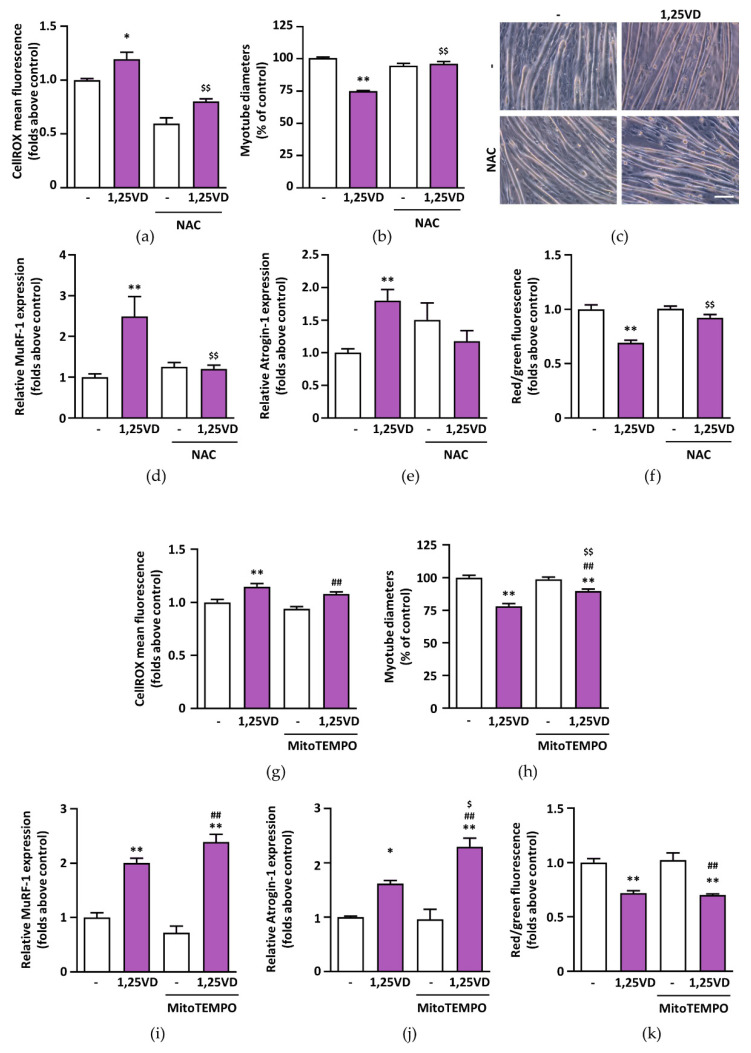
NAC, but not mitoTEMPO, inhibits 1,25VD-induced atrophy in C2C12 myotubes. C2C12 myotubes were treated in serum-free medium with 100 nM 1,25VD in the presence or absence of 5 mM NAC for 24 h. At the end of the treatments, (**a**) cells were stained with CellROX Deep Red reagent to quantify ROS levels and (**b**) myotube diameters were measured with JMicroVision software. (**c**) Phase-contrast representative images of treated myotubes. Scale bar 200 µm. The expression of (**d**) MuRF-1 and (**e**) Atrogin-1, normalized on *Gusb*, was assessed by Real-Time PCR. (**f**) Mitochondrial membrane depolarization was analyzed by JC-1 assay. To assess whether the mitochondrial ROS production was involved in the 1,25VD-induced atrophy, differentiated C2C12 myotubes were treated for 24 h in serum-free medium with 100 nM 1,25VD with or without 10 µM mitoTEMPO and (**g**) ROS levels, (**h**) myotube diameters, (**i**,**j**) atrogene expression, and (**k**) mitochondrial depolarization were analyzed as described above. Data are presented as means ± SEM of three independent experiments. * *p* < 0.05, ** *p* < 0.01 compared to vehicle-treated cells; $ *p* < 0.05; $$ *p* < 0.01 compared to 1,25VD-treated cells; ## *p* < 0.01 compared to mitoTEMPO-treated cells.

To understand the impact of ROS on 1,25VD-induced atrophy, we cotreated C2C12 myotubes with the antioxidant N-acetylcysteine (NAC). Besides abolishing 1,25VD-induced ROS production (Figure 7a), NAC completely abrogated the atrophic effect of 1,25VD, seen as myotube diameter reduction (Figure 7b,c) and atrogene induction (Figure 7d,e), as well as mitochondria depolarization (Figure 7f), indicating that oxidative stress is the main trigger of the atrophy induced by 1,25VD in C2C12 myotubes. On the other hand, treatment with mitoTEMPO, a specific scavenger of mitochondrial superoxide, had no effect on 1,25VD-induced ROS production (Figure 7g), in agreement with the limited induction of H_2_O_2_ flux (Figure 6c), even though it partially prevented the reduction of myotube diameters caused by 1,25VD (Figure 7h). Nevertheless, this partial protection on myotube size was not accompanied by a rescue in atrogene induction (Figure 7i,j) nor in mitochondrial membrane depolarization (Figure 7k).

### 3.8. In C2C12 Myotubes, 1,25VD-Induced Atrophy Depends on PKC and JNK Activation

**Figure 8:**
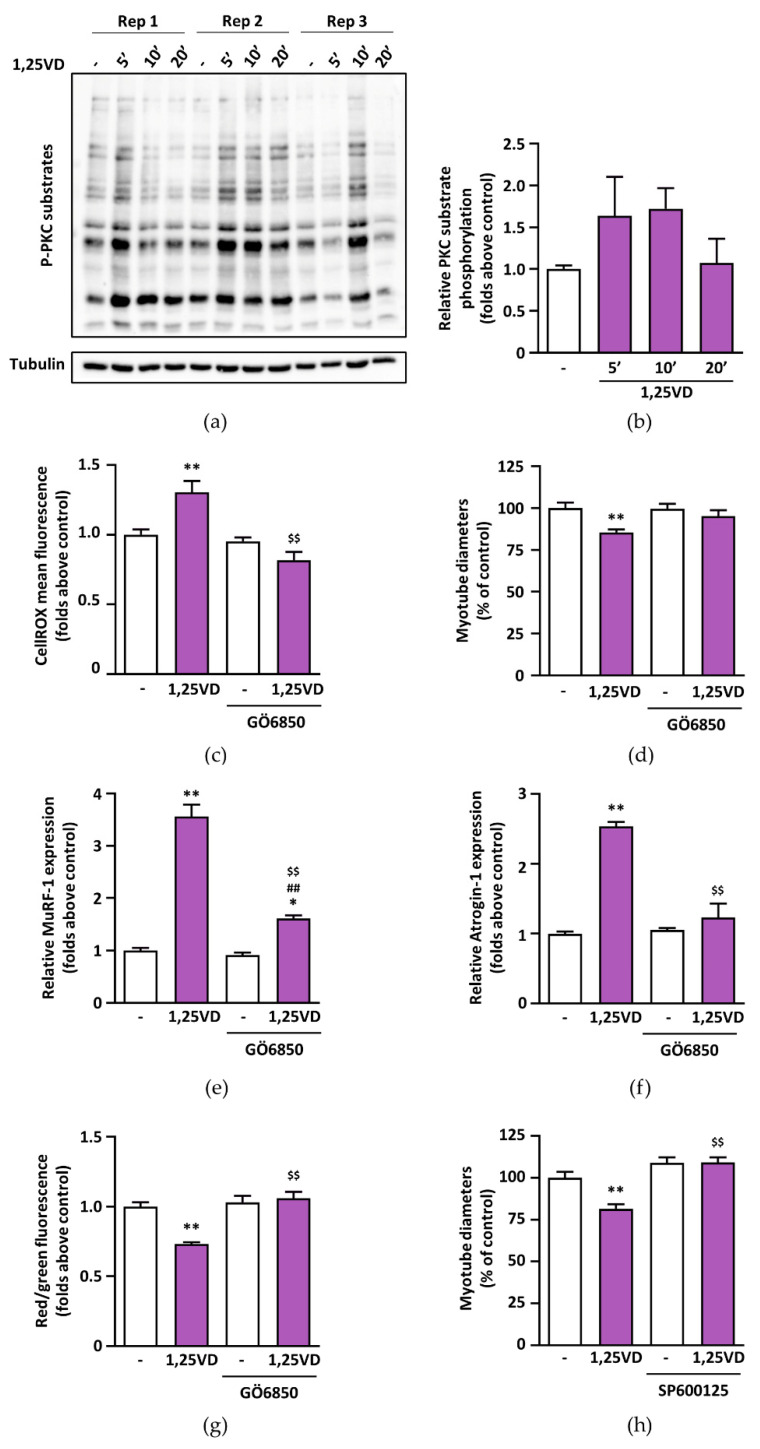
PKC and JNK activation mediates 1,25VD-induced atrophy. C2C12 myotubes were treated in serum-free medium with 100 nM 1,25VD for 5, 10 and 20 min and (**a**) PKC activation was assessed by western blot of its phosphorylated substrates. (**b**) Densitometry of P-PKC substrates normalized on tubulin. To investigate whether PKC is involved in 1,25VD-induced atrophy, C2C12 myotubes were treated in serum-free medium with 100 nM 1,25VD in the presence or absence of 1 µM GÖ6850, a specific PKC inhibitor, for 24 h. At the end of the treatments, (**c**) ROS levels were evaluated with CellROX Deep Red reagent staining, and (**d**) myotube diameters were measured. (**e**,**f**) Expression of atrogenes, normalized on *Gusb*, was assessed by Real-Time PCR and (**g**) mitochondrial membrane depolarization was analyzed by JC-1 assay. (**h**) C2C12 myotubes were treated for 24 h in serum-free medium with 100 nM 1,25VD in the presence or absence of 1 µM SP600125, a specific JNK inhibitor. Data are presented as means ± SEM of three independent experiments. * *p* < 0.05, ** *p* < 0.01 compared to vehicle-treated cells; $ *p* < 0.05; $$ *p* < 0.01 compared to 1,25VD-treated cells; ## *p* < 0.01 compared to GÖ6850-treated cells.

Beside mitochondria, the main producers of cellular ROS are NAD(P)H oxidases (NOXs), the major isoforms of which are NOX4 and 2 in skeletal muscle [27]. Since PKC can induce the activation of NOX2 [28], we assessed whether the PKC pathway is involved in 1,25VD-induced atrophy. Treatment of C2C12 myotubes with 1,25VD induced a very rapid and transient activation of the PKC pathway, as shown by the phosphorylation of its substrates (Figure 8a,b). Intriguingly, the suppression of this pathway by means of a specific PKC inhibitor abolished 1,25VD-induced production of ROS, reduction of myotube diameters, induction of atrogenes, and depolarization of mitochondria (Figure 8c–h). 

Likewise, the inhibition of JNK, a known downstream mediator of ROS-induced atrophy [29,30], abolished the effect of 1,25VD on myotube size (Figure 8g), suggesting that 1,25VD induces atrophy via a PKC-ROS-JNK pathway. 

## 4. Discussion

Vitamin D, especially in the form of 1,25VD, is often regarded as being endowed with antioxidant capacity which allows it to mitigate ROS production and prevent oxidative stress in skeletal muscle [31,32]. However, in this work, we demonstrated that 1,25VD induces production of ROS in C2C12 myotubes that, in turn, cause the atrophic phenotype in these cells.

The notion that mitochondrial dysfunction can lead to muscle wasting [33] applied to our model of 1,25VD-induced atrophy [18], prompted us to assess if 1,25VD impinged mitochondrial function in C2C12 myotubes. To our surprise, besides a strong depolarization of mitochondrial membranes (Figure 1), 1,25VD failed to elicit any significant effect on myotube mitochondria in intact cells (Figure 2 and Figure 4). Only in permeabilized cells, i.e., a system that resembles the analysis of isolated mitochondria, we did observe a decrease in the maximal oxidative phosphorylation capacity of complex I (Figure 3c). Therefore, the drop in mitochondrial membrane potential could hardly be a direct consequence of mitochondrial damage. Since it has been suggested that ROS production is upstream of mitochondrial depolarization [34], we assessed the effect of 1,25VD on ROS production. In line with the limited effects on mitochondrial respiration, 1,25VD did not induce mitochondria-generated ROS (Figure 6c,d). Nevertheless, the intensity of the fluorescence of the probe measuring ROS production dramatically increased after 1,25VD treatment (Figure 6a,b), and the use of a generic antioxidant such as NAC completely prevented the atrophy associated with 1,25VD treatment (Figure 7a–f), suggesting that 1,25VD induces the generation of ROS from cellular sources other than mitochondria. Consistently, the inhibition of mitochondrial ROS was not equally effective in protecting C2C12 myotubes from 1,25VD-induced atrophy (Figure 7g–k). Despite the fact that mitochondria are often considered the most relevant ROS source, in skeletal muscle cells, NAD(P)H oxidases (NOXs) have recently emerged as an equally important source of ROS able to crosstalk with mitochondria to exacerbate ROS production [27]. Although the specific role and activation pathways of each NOX isoform in skeletal muscle have been only partially elucidated, one possible mechanism of activation of NOX2 is mediated by activation of classical PKCs [28]. The involvement of PKC in 1,25VD-mediated MAP kinase activation in chicken myoblasts [35] and the tendency of 1,25VD to phosphorylate PKC substrates in C2C12 myotubes (Figure 8a,b) led us to speculate that 1,25VD-induced ROS production depends on PKC activation. In turn, ROS can lead to muscle atrophy via activation of JNK [29,30]. The hypothesis that 1,25VD-induced atrophy could depend on the activation of a PKC-ROS-JNK pathway is supported by the rescue of the atrophic phenotype when myotubes were treated with specific PKC and JNK inhibitors (Figure 8c–h).

We previously demonstrated that 1,25VD-induced atrophy was accompanied by a block of the autophagic flux in C2C12 myotubes [18]. Here, we observed that the blocking of the autophagic flux involves mitochondria as well (Figure 5). We speculate that 1,25VD-induced mitochondria depolarization is a trigger for mitophagy [36]. However, whether 1,25VD-induced block of mitophagy is under direct control of ROS is still to be determined. Altogether, these data deepen our knowledge of the mechanisms underlying 1,25VD-induced atrophy in C2C12 myoblasts.

The contrasting conclusions of our work compared with those of previously published studies probably depend, in large part, on differences in the methodologies or cells used. Indeed, the majority of the works reporting positive activity of 1,25VD on mitochondria were performed on undifferentiated myoblasts [13,16], while our data, as well as previously published data demonstrating the atrophic activity of 1,25VD [18], were obtained on differentiated myotubes, a cellular model which is closer than myoblasts to adult muscle fibers [19]. Indeed, the content, network shape, and function of mitochondria dramatically change with differentiation from myoblasts to myotubes [37]. Accordingly, when analyzing the OCR in myoblasts treated with 1,25VD, we found a moderate increase in the maximal mitochondrial respiration (data not shown), in accordance with previously reported findings [13]. In addition to the basal different content and activity of mitochondria in myoblasts vs. myotubes, we reported, upon 1,25VD treatment, an apparent lack of VDR-RXR nuclear translocation in myotubes compared to myoblasts [19]. Since proper VDR function is required for 1,25VD effects on myoblasts mitochondrial activity [15], this could be a further mechanism underlying the differences in the effects of 1,25VD on myotubes compared to myoblasts.

On the other hand, the effects of 1,25VD upstream metabolites VD3 and 25VD on mitochondria are less straightforward. As both VD3 and 25VD own a protective activity on C2C12 myotubes in the presence of atrophic stimuli [18,19], it is possible to envision that more striking effects on mitochondrial metabolism could be detected in an atrophic context. The differences between VD3 and 25VD in some assays (Figure 1b and Figure 2b,c) may point to the fact that VD3 is not converted to 25VD in C2C12 myotubes, and that it probably acts through different mechanisms [19]. Nevertheless, both VD3 and 25VD increased mitochondrial content (Figure 4a) and reduced basal ROS (Figure 6a,b), suggesting that their anti-atrophic properties can depend, in part, on antioxidant activity. 

## 5. Conclusions

Vitamin D is usually credited with having antioxidant properties, but our data show that different metabolites can have different effects on ROS production. Therefore, we postulate that the efficacy of vitamin D supplementation in vivo depends on the balance between atrophic (1,25VD) and protective (VD3 and 25VD) metabolites, and that the altered expression of specific vitamin D hydroxylases in aging vs. other pathologies affecting skeletal muscle homeostasis might swing the balance. However, further studies will be necessary to assess the potentiality and limits of vitamin D supplementation to prevent skeletal muscle loss in aging and pathological states.

## Data Availability

The data presented in this study are available in this manuscript. Raw data are available upon substantiated request [data presented in this work are not the kind that need to be made available on public databases].
